# Theory and Measurement of Signal-to-Noise Ratio in Continuous-Wave Noise Radar

**DOI:** 10.3390/s18051445

**Published:** 2018-05-06

**Authors:** Bronisław Stec, Waldemar Susek

**Affiliations:** Faculty of Electronics, Military University of Technology, Gen. Urbanowicz St. No 2, 00-908 Warsaw, Poland; bronislaw.stec@wat.edu.pl

**Keywords:** radar receiver, correlation, radar clutter, signal-to-noise ratio, radar measurements

## Abstract

Determination of the signal power-to-noise power ratio on the input and output of reception systems is essential to the estimation of their quality and signal reception capability. This issue is especially important in the case when both signal and noise have the same characteristic as Gaussian white noise. This article considers the problem of how a signal-to-noise ratio is changed as a result of signal processing in the correlation receiver of a noise radar in order to determine the ability to detect weak features in the presence of strong clutter-type interference. These studies concern both theoretical analysis and practical measurements of a noise radar with a digital correlation receiver for 9.2 GHz bandwidth. Firstly, signals participating individually in the correlation process are defined and the terms *signal* and *interference* are ascribed to them. Further studies show that it is possible to distinguish a signal and a noise on the input and output of a correlation receiver, respectively, when all the considered noises are in the form of white noise. Considering the above, a measurement system is designed in which it is possible to represent the actual conditions of noise radar operation and power measurement of a useful noise signal and interference noise signals—in particular the power of an internal leakage signal between a transmitter and a receiver of the noise radar. The proposed measurement stands and the obtained results show that it is possible to optimize with the use of the equipment and not with the complex processing of a noise signal. The radar parameters depend on its prospective application, such as short- and medium-range radar, ground-penetrating radar, and through-the-wall detection radar.

## 1. Introduction

A characteristic of noise radars is the use of random or pseudorandom signals for probing purposes [1]. Until relatively recently, the design of noise radars was hindered by serious practical problems. Due to the absence of sufficiently fast computing platforms, the receivers of this type of radar were mainly constructed with the use of analogue solutions [2,3]. Currently, owing to the increasing availability of specialized high-performance processors and general-purpose platforms such as FPGAs (field-programmable gate arrays) and due to the availability of fast high-resolution analogue-to-digital converters, it has become possible to build noise radars equipped with digital receivers designed for various applications. The subject literature includes research on the use of noise radars in ultra-wideband SAR/ISAR (synthetic aperture radar/inverse synthetic aperture radar) systems [4], to conduct Doppler measurements [5], in anti-collision systems, in polarimetric measurements to detect objects hidden underground [6], and to detect micrometric distance changes, heartbeat, or chest movements in living organisms [3,7]. The use of signal processors with range-Doppler detection for real-time signal processing has increased the functionality of noise radars. The correlation of transmitted and received signals in the baseband can be performed simultaneously in multiple range cells, owing to the application of different reference signal delays in the signal processor. However, compared to the computational requirements of pulse-Doppler radar, noise radar still requires considerably more computational power in order to detect an object. An additional problem is a phenomenon characteristic of continuous-wave noise radars—weak reflections from distant objects masked by strong signals coming from objects near the radar. A noise radar with a continuous wave receives both weak and strong signals simultaneously. The receiver dynamic range is connected with the following phenomena: dynamic range related to target RCS (radar cross section) and dynamic range related to a distance. For instance, for a battlefield radar, the RCS of a human may be 0.5 m^2^, while the RCS of the ground is 1000 m^2^. Therefore, the dynamic range dependent on RCS changes is 33 dB. The dynamic range related to distance is based on the law that a reflected signal’s power reduces with the fourth power of distance. For example, for the shortest (10 m) to the longest (1000 m) distance ratio, the dynamic range is 80 dB. Therefore, it is necessary to ensure a very high reception path with a dynamic range equal to or exceeding 100 dB. An optimal receiver for this type of signal is a correlation receiver. A correlation receiver not only deals with the noise of the receiver’s electronic circuits and components, but also with the noise occurring as a correlation function of noise signals, which is the so-called noise floor. The RMS (root mean square) of the noise signal’s correlation function estimator is not reduced to zero with a higher distance. Therefore, it is transferred to all other distance cells. This results in the emergence of an additional (apart from the self-noise of components and circuits) interference noise source that limits the receiver sensitivity. Recently, a number of studies presenting research reducing clutter with algorithmic methods in the digital correlation processing of noise signals have been published [8,9,10,11,12,13]. This paper discusses the mutual signal power relations in an actual noise radar solution with a range-Doppler digital correlator. This research allows us to determine an achievable limit level of SNR (signal-to-noise ratio) at the output of the correlation detector depending on the SNR at its input for band-limited white noise signals without the use of algorithmic signal processing techniques to reduce the noise floor. The considerations presented in the study concern various noise signals with the same probabilistic characteristics. The signal coming from the analyzed target and the signals coming from other targets, leakage signals, and ground-reflected signals are noise signals with zero mean value and Gaussian distribution of probability density. For this reason, a classic approach to radar system parameters such as receiver sensitivity, SNR, or detection gain requires determination of the signal and the noise in a noise radar both at the input and at the output of the correlation detector. The specific nature of continuous-wave noise radars is that both the power of the noise signal received from the target and the power of the interfering signals depends on the radar transmitter signal power. One exception is the receiver self-noise. The studies which are presented in this paper focus on discovering the causes of the “noise floor” occurrence in the correlative detector output signal. The conducted measurements prove the theoretical considerations, which were sufficiently described in the work of Axelsson [14]. This paper analyzes a more complex case in which, while referring to Reference [14], an interfering signal σ_z_ consists of a signal of internal leakage, antenna leakage, and other signals received by an antenna (e.g., signals reflected from the ground). There is also a reference signal σ^2^_ref_ that is not required to be equal to a received signal σ^2^_x_ as it is in Reference [14].

## 2. Signals in a Noise Radar

**Figure 1:**
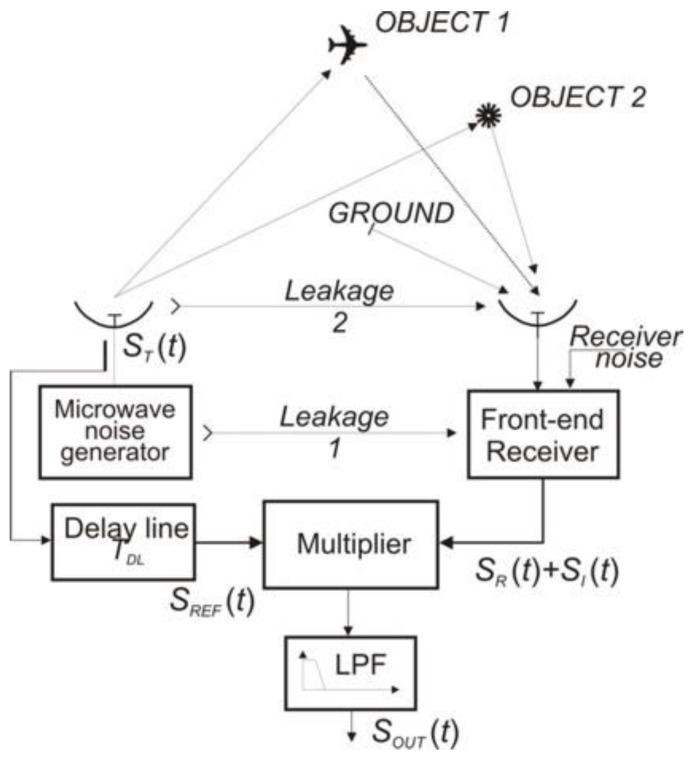
Functional diagram of a noise radar together with the types of the signals occurring in real working conditions. LPF – Low Pass Filter.

Figure 1 shows a simplified functional diagram of a noise radar in combination with existing signal types in real operating conditions. In a correlation receiver, the following signals exist: the desirable signal, which comes from *Object 1* and is indicated in Figure 1 with *S_R_*(*t*); and some other signals indicated with *S_I_*(*t*), which are interfering signals from the viewpoint of signal *S_R_*(*t*). The interference signals include two types of signals: a signal associated with the transmitter signal *S_T_*(*t*) and a signal independent of the transmitter, which is the receiver self-noise *S_N_*_,*R*_(*t*). These signals have the characteristics of random signals and limit the receiver sensitivity.

Signals associated with the radar transmitter noise signal include:

- *leakage 1*—internal leakage of a signal from transmitter circuits to the receiver circuits with the power P_LC_.
- *leakage 2—*leakage of a signal between the transmitting and receiving antennas with the power P_LA_.
- *interfering signal—*signal from *Object 2* with the power P_R2_ and signals reflected from the ground and other elements on its surface at different distances from the radar with the total power P_C_ a so-called clutter.

In order to simplify the analysis of the results of the noise signal correlation processing, the number of signals involved in the process of correlation with reference signal *S_REF_*(*t*) was reduced to two: *S_R_*(*t*), the signal from *Object 1*; and *S_I_*(*t*), the interfering signal. The power of the interfering noise signal can be defined as:

(1) $$P_{I}=P_{LC}+P_{LA}+P_{R2}+P_{C}+P_{N,R}=P_{N,T}+P_{N,R}$$

The components of the signal *S_N_*_,*T*_(*t*) are sources of correlation peaks for the delay times other than the delay time of the target in the process of correlation with the reference signal. However, they constitute a significant interference for the signal reflected from Object 1 due to the additive residue of the correlation function of these signals.

## 3. Noise Signal Correlation Function

The processing of the noise signals in a noise radar is related to the time segments of two realizations *x*(*t*) and *y*(*t*) of two stationary and ergodic stochastic processes *X*(*t*) and *Y*(*t*) [15]. The physical meaning of these signals refers to a signal reflected from the target and a reference signal or a reference signal and an interference signal. Two process segments called the noise signals of a specified duration are considered in this paper. In noise radar systems, the mean values of signals *x*(*t*) and *y*(*t*) are equal to zero. In the case of continuous signals existing in a specified time interval (*T* + *τ*), the estimator ${\hat{R}}_{xy}\left(\tau \right)$ of their mutual correlation function (cross-correlation) can be represented by the following Equation (2) [16].

(2) $${\hat{R}}_{xy}\left(\tau \right)=\frac{1}{T}\int_{0}^{T}x(t)y(t-\tau )dt,\;0\leq \tau <T.$$

**Figure 2:**
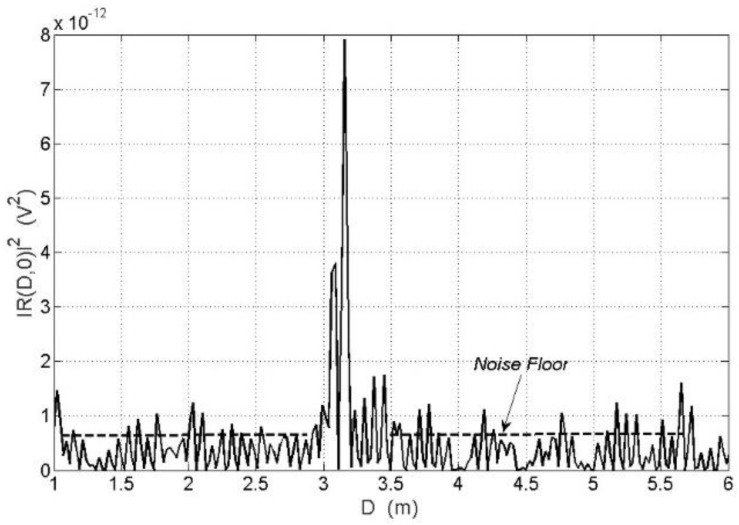
Real plot of the correlation function module square for *D_o_* = 3.2 m, *v* = 0, *B* = 1 GHz, *T* = 160 μs.

At a large time distance from the correlation peak, the correlation function does not decrease to zero but maintains a residual value called a noise floor of the correlation function illustrated in Figure 2, which is of special importance to the noise radar sensitivity and range.

Therefore, it is important to determine the values of the correlation function outside of the correlation peak.

### 3.1. The Variance of the Random Signal Correlation Function Estimator

It should be noted that for *τ* = 0, Expression (2) becomes an estimator of the RMS value of the realization *x*(*t*) or *y*(*t*). By definition, the expected value of the cross-correlation function estimator (2) is determined by Equation (3) [16]:

(3) $$E\left[{\hat{R}}_{xy}\left(\tau \right)\right]=\frac{1}{T}{\int_{0}^{T}E\left[x(t)y(t-\tau )\right]}^{}dt=\frac{1}{T}{\int_{0}^{T}R_{xy}\left(\tau \right)}^{}dt=R_{xy}\left(\tau \right).$$

Therefore, ${\hat{R}}_{xy}\left(\tau \right)$ is an unbiased estimator of the cross-correlation function *R_xy_*(*τ*) regardless of the signal duration *T*. Therefore, the mean squared error of estimator ${\hat{R}}_{xy}\left(\tau \right)$ is equal to its variance according to Equation (4) below [16]:

(4) $$D^{2}\left[{\hat{R}}_{xy}\left(\tau \right)\right]=\frac{1}{T}{\int_{0}^{T}\int_{0}^{T}\left(E\left[x\left(u\right)y\left(u+\tau \right)x\left(\upsilon \right)y\left(\upsilon +\tau \right)\right]-R_{xy}^{2}\left(\tau \right)\right)}^{}d\upsilon du.$$

For further analysis, it was assumed that two stochastic processes X(*t*) and Y(*t*), which are jointly and separately normal processes, would be subjected to correlation processing. Therefore, for a normal random process with zero mean value, variance (4) is expressed by Equation (5) below [16]:

(5) $$D^{2}\left[{\hat{R}}_{xy}\left(\tau \right)\right]\approx \frac{1}{T}{\int_{-\infty }^{\infty }\left(R_{x}\left(\psi \right)R_{y}\left(\psi \right)+R_{xy}\left(\psi +\tau \right)R_{yx}\left(\psi -\tau \right)\right)}^{}d\psi ,$$

where *ψ* = *υ − u*, d*ψ* = d*υ*, *x*(*t*) and *y*(*t*) are the realization of the white noise with a limited frequency band equal to *B* and sufficiently long duration *T*.

The variance of the estimator of the cross-correlation function of these realizations determined on the basis of (5) is shown in Equation (6) below [16]:

(6) $$D^{2}\left[{\hat{R}}_{xy}\left(\tau \right)\right]\approx \frac{1}{2BT}\left[R_{x}\left(0\right)R_{y}\left(0\right)+R_{{}_{xy}}^{2}\left(\tau \right)\right].$$

### 3.2. Noise at the Correlation Detector Output

**Figure 3:**
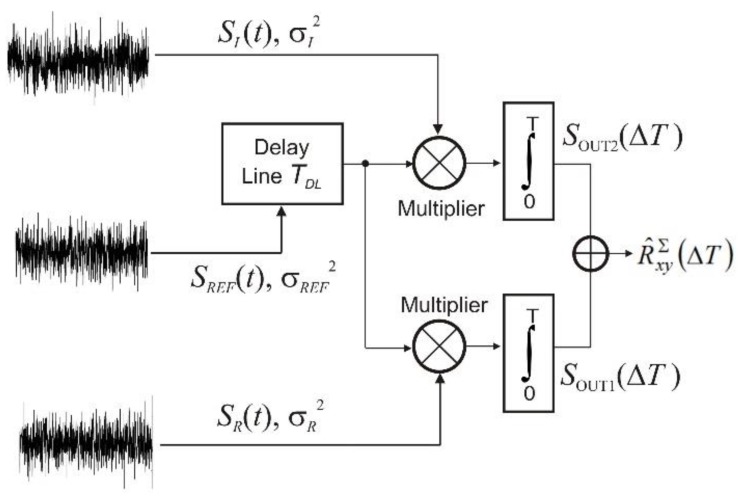
Illustration of signal processing in a noise radar.

The analysis of signal correlation processing in the noise radar was based on the following assumptions, depicted in Figure 3.

There are three input noise signals with duration *T*, zero mean value, and Gaussian distribution of probability density function, which occupy band *B*; that is, a signal received from target *S_R_*(*t*) with variance σ*_R_*^2^, reference signal *S_REF_*(*t*) with variance σ*_REF_*^2^, and interfering signal *S_I_*(*t*) with variance σ*_I_*^2^. As a result of the correlation processing in the noise radar receiver, these signals correlate with each other as follows: the reference signal with the signal received from the target results in the output signal *S*_OUT1_(Δ*T*), which is the estimate of the cross-correlation function *R_xy_*_,*I*_(Δ*T*) of these signals. The reference signal with the interfering signal results in the output signal *S*_OUT2_(Δ*T*), which is the estimate of the cross-correlation function *R_xy_*_,*II*_(Δ*T*) of the reference signal and the interfering signal. In fact, one correlation processing is performed, which results in the determination of the estimate of the cross-correlation function *R_xy_^∑^*(Δ*T*). This estimate is identical to the sum of the estimates.

(7) $${\hat{R}}_{xy}^{\Sigma }\left(\Delta T\right)=S_{OUT1}\left(\Delta T\right)+S_{OUT2}\left(\Delta T\right),$$

where Δ*T* = *T_O_ − T_DL_*, *T_O_* = 2*D_O_*/c, *D_O_* is the distance from radar to an object, and *T_DL_* is the time delay of the delay line.

Based on definition (5), variance *R_xy_^∑^*(Δ*T*) can be described with Equation (8) below:

(8) $$D^{2}\left[{\hat{R}}_{xy}^{\Sigma }\left(\Delta T\right)\right]=D^{2}\left[S_{OUT1}\left(\Delta T\right)\right]+D^{2}\left[S_{OUT2}\left(\Delta T\right)\right].$$

The variance of estimate ${\hat{R}}_{xy}^{\Sigma }\left(\Delta T\right)$ is equal to the sum of variances of estimates *S_OUT_*_1_(Δ*T*) and *S_OUT_*_2_(Δ*T*). The relationship between the parameters of the input and output signals of the correlation detector are as follows: *R_R_*(0) = σ*_R_*^2^ is the variance of the noise signal from the target equal to the power *P_R_*_,*IN*_; *R_I_*(0) = σ*_I_*^2^ is the variance of the signal from the interference equal to the power *P_I_*; *R_REF_*(0) = σ*_REF_*^2^ is the variance of the reference signal equal to the power *P_REF_*. All powers are measured at 1 Ω resistance. On the other hand, the voltage at the correlator output for Δ*T* = 0 is determined by Equation (9) below:

(9) $$S_{OUT1}\left(0\right)=\alpha \sqrt{\sigma_{R}^{2}\sigma_{REF}^{2}},$$

where α (V/W) is the coefficient of transmission of the correlation detector.

By inserting Equation (6) in Equation (8), it is possible to determine the variance of the correlation function estimate, which is expressed by the following formula:

(10) $$D^{2}\left[{\hat{R}}_{xy}^{\Sigma }\left(\Delta T\right)\right]\approx \frac{\alpha^{2}}{2BT}\left(\sigma_{R}^{2}\sigma_{REF}^{2}+R_{xy,I}^{2}\left(\Delta T\right)\right)+\frac{\alpha^{2}}{2BT}\left(\sigma_{REF}^{2}\sigma_{I}^{2}+R_{xy,II}^{2}\left(\Delta T\right)\right).$$

The source of all signals except for receiver self-noise in a noise radar is its transmitter. These signals are therefore correlated, and are in the form of Gaussian white noise. The signal power on the correlator output, at mutual correlation of, for example, a signal reflected from the target and a reference signal for Δ*T* ≠ 0 is smaller than this signal power by *BT* for Δ*T* = 0. This results in the fact that for *BT* = 10, for example, component *R*^2^*_xy_*_,*I*_(ΔT ≠ 0) is only 10% of σ*_R_*^2^σ*_REF_*^2^. A similar situation is seen in the case of the second component of Equation (10). Therefore, for a sufficiently high value of *BT*, the values of factors *R_xy_*_,*I*_^2^(Δ*T*) and *R_xy_*_,*II*_^2^(Δ*T*) are negligible compared to the values of the corresponding products (σ*_R_*^2^σ*_REF_*^2^) and (σ*_REF_*^2^σ*_I_*^2^). Considering the above, Equation (10) takes the following form:

(11) $$D^{2}\left[{\hat{R}}_{xy}^{\Sigma }\left(\Delta T\neq 0\right)\right]=P_{N,OUT}\approx \frac{\alpha^{2}}{2BT}\left(P_{R,IN}P_{REF}+P_{REF}P_{I}\right).$$

Equation (11) describes the variance of the cross-correlation function estimator of the signals occurring in the noise radar. Equation (11) also allows the determination of correlator output noise power P_N,OUT_, or the noise floor, which is shown in Figure 2. The power of the correlator output noise is equal to the variance of the correlation function estimator for Δ*T*, which significantly differs from zero. It is also possible to determine the PNFR (peak-to-noise floor ratio) as a ratio of the power of the output signal *S_OUT_*_1_(Δ*T*) for Δ*T* = 0, to the variance of estimator ${\hat{R}}_{xy}^{\Sigma }\left(\Delta T\right)$ for Δ*T* ≠ 0.

(12) $$\mathrm{PNFR}=\frac{{\left|S_{OUT1}\left(\Delta T=0\right)\right|}^{2}}{D^{2}{\left[{\hat{R}}_{xy}^{\Sigma }\left(\Delta T\neq 0\right)\right]}^{{}^{}}}\approx \frac{\sigma_{R}^{2}\sigma_{REF}^{2}}{\frac{1}{2BT}{\left(\sigma_{R}^{2}\sigma_{REF}^{2}+\sigma_{REF}^{2}\sigma_{I}^{2}\right)}^{}},$$

where the numerator of Equation (12) describes the power of the correlator output signal. The denominator of (12) describes the power of the correlator output noise, or the so-called noise floor.

## 4. Experimental Measurements

**Figure 4:**
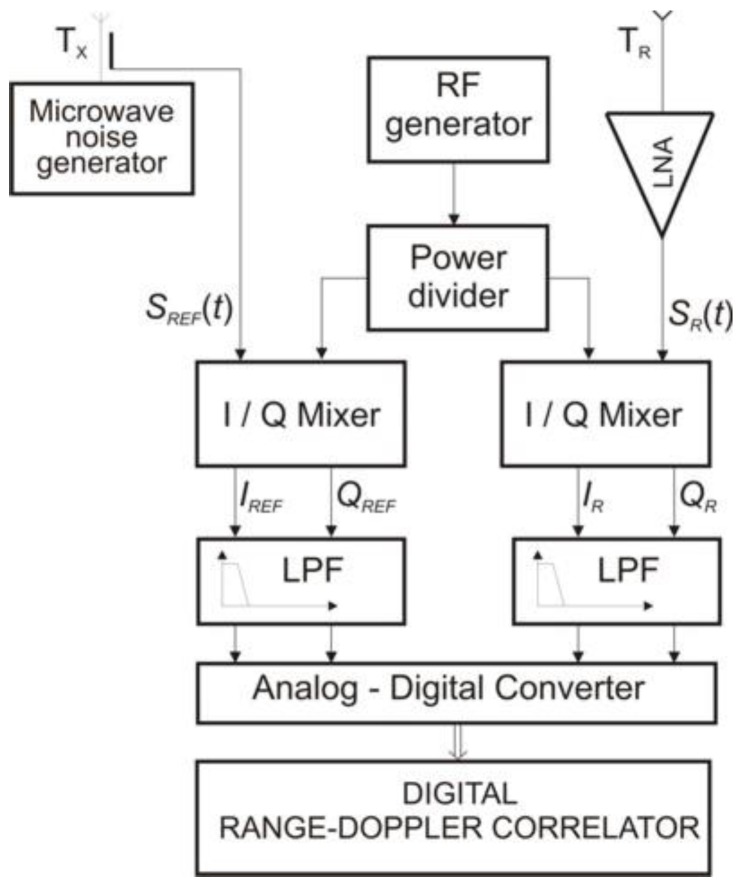
The block diagram of the noise radar with a digital range-Doppler correlator.

Measurements were performed on the design solution of a noise radar with a continuous wave signal. The noise radar block diagram is shown in Figure 4.

Basic noise radar parameters include the maximum transmitter power *P_T_* = 10 dBm, the noise signal bandwidth *B_RF_* = 150 MHz, center frequency *f*_0_ = 9.2 GHz, and integration time *T* = 1 ms. In real systems, the main measurement problem is the separation of the noise signal coming from the target from other noise signals such as internal leakage, antenna leakage, and other signals received by an antenna. This includes signals reflected from the ground. These signals determine the noise power at the output of the digital correlator.

### 4.1. Measuring System

**Figure 5:**
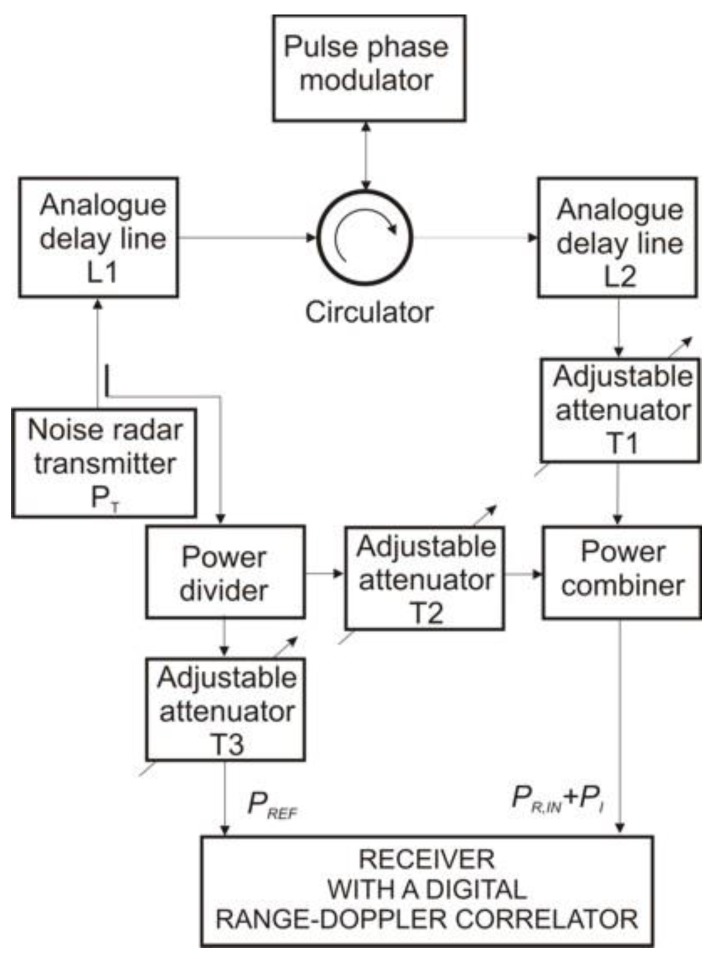
Measuring system for noise radar signals measurement.

Figure 5 shows the measuring system used to measure the power of the noise radar signals. The signal path was implemented using a coaxial transmission line with length L1 to connect a noise radar transmitter with a microwave pulse phase modulator. The phase modulator was used together with a circulator to imitate a moving object.

The phase modulator introduced a Doppler shift corresponding to the specified radial speed of the object into the signal path. The second line L2 with the same length as L1 represents the signal path. To verify Equation (12), the pulse phase modulator performed a full reflection of the power supplied to it from the transmitter. The adjustable attenuator T1 made it possible to change the value of signal *S_R_*_,*IN*_(t), which reached the receiver signal input through a power combiner. Reference signal *S_REF_*(t) was transmitted to the receiver reference input through a power divider. Part of the signal from the noise transmitter was transferred to the combiner through the power divider and adjustable attenuator T2, and then, to the receiver signal input. This signal represents the sum of the following signals: internal leakage, leakage between antennas, and ground-reflected signals specified in Equation (1). At the receiver input, there was a known value of noise signal *P_R_*_,*IN*_ coming from the target and the value of interference power *P_I_*. The lengths of the transmission lines connecting the noise generator to the correlation receiver in the reception and reference paths were negligible in relation to the length of the lines L1 and L2. The power of the receiver self-noise included in the power of interference noise according to Equation (1) was calculated on the basis receiver noise ratio measurement.

### 4.2. Measurement Results

**Figure 6:**
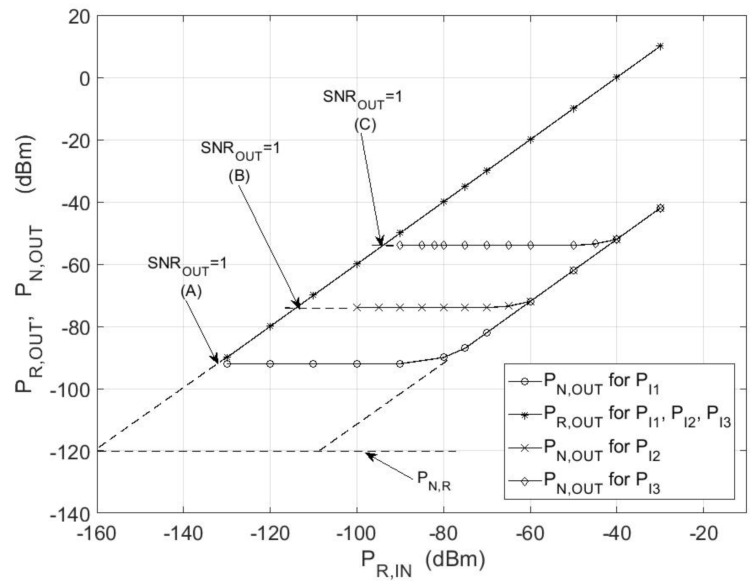
The plot of signal power transmittance for various interfering signals for *B_RF_* = 150 MHz, *T* = 1 ms, *f*_0_ = 9.2 GHz. SNR_OUT_: output signal-to-noise ratio.

Figure 6 shows three pairs of characteristics: *P_R_*_,*OUT*_ and *P_N_*_,*OUT*_ of the correlation receiver output for Δ*T* = 0 as a function of input signal power *P_R_*_,*IN*_. These characteristics were plotted for three different values of interfering signal power *P_I_*. The change in the value of power *P_R_*_,*IN*_ was implemented using an adjustable attenuator T1. The plot of the measured characteristics *P_R_*_,*OUT*_ = *f*(*P_R_*_,*IN*_) was consistent with the equation below:

(13) $$P_{R,OUT}=\alpha^{2}k_{T1}k_{T3}\sigma_{T}^{4}=\alpha^{2}P_{R,IN}P_{REF},$$

where *k_T_*_1_ and *k_T_*_3_ are coefficients of the transmittance of the noise generator signal power for the receiving channel and the reference channel, respectively, and σ^2^*_T_* is the variance of the noise generator signal.

Three characteristics *P_R_*_,*OUT*_ were plotted for different values of power *P_I_* starting at points (A), (B), and (C), marked in Figure 6, and partially overlapped with each other. These points correspond to the unit value of the SNR at the correlator output for *P_I_*_1_ = *P_LC_*+*P_N_*_,*R*_, *P_I_*_2_ = *P_I_*_1_+1nW, and *P_I_*_3_ = *P_I_*_1_ + 0.1 μW. Figure 6 also includes *P_N_*_,*OUT*_ measured under the same conditions, which partially overlapped as well. This is similar to the signal characteristics. In this case, output noise power *P_N_*_,*OUT*_ is demonstrated by a broken curve described by the following Equation (14):

(14) $$P_{N,OUT}=\alpha^{2}\frac{k_{T1}k_{T3}\sigma_{T}^{4}+k_{T3}\sigma_{T}^{2}\left(k_{T2}\sigma_{T}^{2}+\sigma_{N,R}^{2}\right)}{B_{RF}T}=\alpha^{2}\frac{P_{R,IN}P_{REF}+P_{REF}P_{I}}{B_{RF}T}.$$

If signal power *P_R_*_,*IN*_ is much less than the interfering signal power *P_I_*, then (14) takes the form below:

(15) $$P_{N,OUT}≅\frac{\alpha^{2}}{B_{RF}T}P_{REF}P_{I}.$$

As it follows from Equation (15), *P_N_*_,*OUT*_ does not depend on the power of the input signal coming from the target. In Figure 6, this situation is represented by a flat part in the characteristics with the level depending only on the value of the interfering signal power *P_I_*. *P_R_*_,*IN*_ ≫ *P_I_* (15) is simplified to the form below:

(16) $$P_{N,OUT}≅\frac{\alpha^{2}}{B_{RF}T}P_{R,IN}P_{REF}.$$

The output noise power is proportional to the input signal power and is inversely proportional to the *B_RF_T*. For *P_R_*_,*IN*_ = const, the difference in the signal power levels and noise power levels at the correlator output, expressed in dB, is equal to value 10log(*B_RF_T*).

The application of the proposed measurement system presented in Figure 5 enabled the separation of the useful signal from the interference signals, even though all of the signals were in the form of white noise and occurred together. Based on the results presented in Figure 6, it is possible to determine the value of internal leakage signal power for a given noise radar structure. This signal, along with the antenna leakage signal and signals reflected from other objects (e.g., ground), influences the level of noise on the correlative detector output. While measuring the total power of noise on the correlator output, it is not possible to determine the measurement corresponding to the power of the input signal, which is similar in the case of output SNR. On the correlator output, the signal-to-noise ratio may adopt the same value for different levels of input signal power (e.g., points A and B in Figure 6).

**Figure 7:**
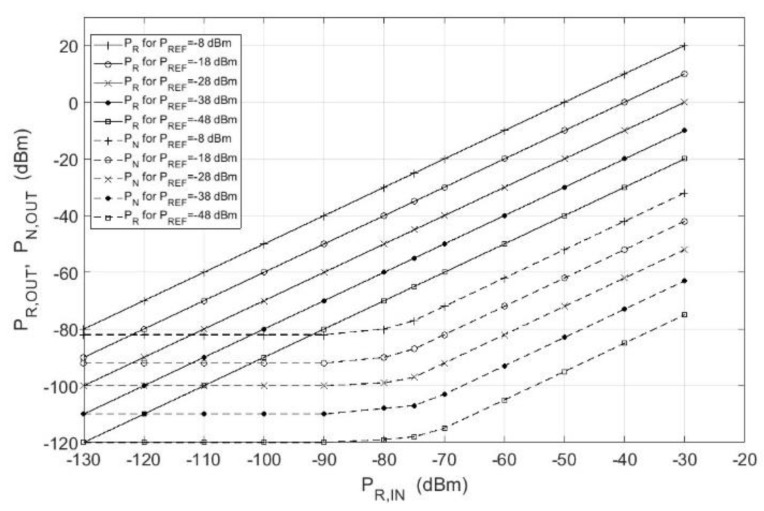
The plot of the signals power transmittance for various reference signals for *B_RF_* = 150 MHz, *T* = 1 ms, *f*_0_ = 9.2 GHz.

Figure 7 shows the impact of the reference channel signal power *P_REF_* on *P_R_*_,*OUT*_ and *P_N_*_,*OUT*_ at the correlation receiver output as a function of the input signal power.

Correspondingly, all the characteristics presented the same shape as those in Figure 6. The value of the reference channel power *P_REF_* decided only the increase in the output power of the signals. However, *SNR_OUT_* for specified *P_R_*_,*IN*_ remained unchanged.

**Figure 8:**
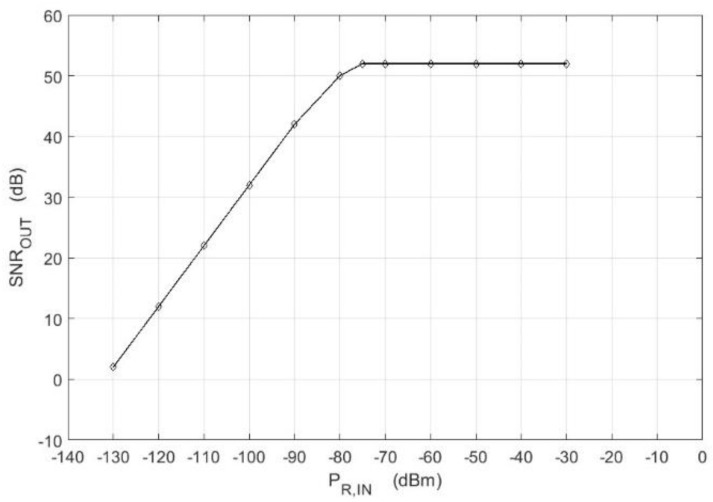
The plot of the SNR at the correlator output for *P_REF_* = −18 dBm, *B*_RF_ = 150 MHz, *T* = 1 ms, *f*_0_ = 9.2 GHz.

One of the basic characteristics of a correlation receiver that describes the SNR as a function of input signal power is shown in Figure 8.

The characteristic was plotted for the interference power equal to *P_I_*, which corresponds to the sum of internal leakage power *P_LC_* and receiver self-noise power *P_N_*_,*R*_. According to Equation (12), *SNR*_OUT_ ratio as a function of *SNR*_IN_ is expressed in Equation (17) below:

(17) $$SNR_{OUT}=\frac{B_{RF}T}{1+SNR_{IN}^{-1}}.$$

It can be observed that Equation (17) takes the following form for *SNR_IN_* << 1:

(18) $$SNR_{OUT}≅B_{RF}T\cdot SNR_{IN}.$$

*SNR*_OUT_ is directly proportional to the value of the *SNR* at the input with a factor of proportionality equal to *B*_RF_*T*. On the other hand, for *SNR_IN_* ≫ 1, (17) takes the form below:

(19) $$SNR_{OUT}≅B_{RF}T.$$

Then, the SNR at the output does not depend on the input signal power and tends to be equal to a constant value of *B*_RF_*T.* The above analytical conclusions are consistent with the measurement results shown in Figure 8.

## 5. Conclusions

The proposed measurement system allows us to determine the relation between output SNR and input signal power, and therefore to optimize the noise radar parameters depending on the desired application. This system allows for the separation of random signals with the same probabilistic characteristics into signals that can be termed *signal* and *noise* at the input and output of the correlation detector.

The measurements of the signal power and the noise power at the correlation receiver output fully support the theoretical relations expressed by Equation (12).

It is important to note that by using the experimental measurements shown in Figure 6, it is possible to determine the values of the noise radar. This includes basic parameters such as transmitter power, operating band, integration time, maximum range, reference signal power, and permissible values of internal leakage and antenna leakage.

Recently, an intensive research study was conducted to reduce the noise floor level (i.e., to reduce the interfering signal power *P_I_* defined by Equation (1)). This reduction can be achieved through a hardware solution by minimizing powers *P_LC_* and *P_LA_*, as well as through signal processing.
